# Socket preservation with and without lovastatin in combination with synthetic bone graft substitute: A double-blind clinical trial

**DOI:** 10.34172/japid.026.3919

**Published:** 2026-02-02

**Authors:** Amirhossein Farahmand, Behzad Houshmand, Amir Reza Shirazi, Yassin Attar Zadeh, Soheil Taghavi Namin, Maryam Zohary, Mohammad Reza Sadeghi

**Affiliations:** ^1^Faculty of Dentistry, Tehran Islamic Azad University of Medical Sciences, Tehran, Iran; ^2^School of Dentistry, Shahid Beheshti University of Medical Sciences, Tehran, Iran; ^3^Private Practice, Tehran, Iran; ^4^Department of Periodontology, Shahed Dental School, Shahed University, Tehran, Iran

**Keywords:** Extraction, Histomorphometry, Lovastatin, Socket preservation, Synthetic bone graft

## Abstract

**Background.:**

The present study examined how bone improvement techniques, particularly the use of lovastatin with ridge preservation strategies, affect bone maintenance after tooth extraction. The goal of these strategies is to potentially decrease bone resorption and enhance the quality of alveolar bone, while also taking into account the associated risks of using high doses of statins.

**Methods.:**

Twenty healthy patients undergoing bone grafting after tooth extraction in the anterior region of the maxilla were randomly divided into groups A and B. After non-invasive extraction, group A received a synthetic bone graft substitute combined with 10 mg of lovastatin, while group B received a synthetic bone graft alone. The graft was placed in the tooth socket and covered with a collagen cone for support. After nine months, a histomorphometric analysis assessed horizontal changes, using a clipper in the area compared to pre-treatment clinical measurements (*P*≤0.05).

**Results.:**

The study examined 40 areas and found that the bone formation rate was 51.69% for those receiving only a synthetic bone graft substitute. When combined with 10 mg of lovastatin, the bone formation rate increased to 60.79%, indicating a significant improvement. The group receiving only the synthetic graft had an average of 1.70 mm leftover particles, while the lovastatin group had only 0.4844 mm particles, suggesting that lovastatin reduced residual material. Additionally, the lovastatin group exhibited a smaller ridge width and no signs of inflammation or foreign body reaction. In contrast, the group receiving only Nanobone showed inflammatory responses, primarily from mononuclear cells.

**Conclusion.:**

According to the results, lovastatin shows promise in enhancing osteogenesis, narrowing the ridge, and decreasing residual connective tissue, while avoiding inflammation and foreign body reactions.

The study was registered on clinicaltrials.gov under the ID NCT03981601.

## Introduction

 Following tooth extraction, alveolar ridge remodeling inevitably results in both vertical and horizontal tissue resorption.^1^ This dimensional alteration presents notable challenges, particularly in the esthetically sensitive anterior maxilla.^2^ Maintaining the integrity of alveolar bone architecture after extraction is paramount for achieving predictable aesthetic and functional outcomes in implant-supported prosthetic rehabilitation.^3^ Alveolar ridge remodeling following tooth removal typically results in dimensional reductions in both vertical and horizontal planes.^4^ Following tooth extraction without socket preservation, clinical investigations indicate a mean vertical bone resorption ranging from 0.7 to 1.5 mm and a horizontal bone resorption of 4.0 to 4.5 mm at six months.^5^ Future alveolar ridge augmentation during implant placement is less necessary due to the reduction in tissue loss, leading to simpler implant surgical procedures.^6‒8^ In the long term, a reduction in alveolar bone volume and the collapse of soft tissue can lead to functional loss, making it difficult to place dental implants and complicating conventional prosthetic treatments.^9^ In addition, socket preservation procedures use a variety of surgical techniques and grafting materials.^10^ Additionally, various grafting materials, such as autologous grafts, allografts, xenografts, and alloplast, have been placed in the alveolar socket right after tooth extraction.^11^ Additionally, the reduction in the dimensions of the alveolar ridge cannot be fully prevented, regardless of the graft material used.^12^ Moreover, these materials have certain disadvantages, including the possibility of disease transmission, high costs, and a limited ability to promote bone growth.^13,14^ The proliferation of osteoblasts is more effectively enhanced by Nanobone, which is made of nano-crystalline hydroxyapatite, than by deproteinized bovine bone minerals.^15,16^ Statins can also significantly impact anti-inflammatory and antioxidant effects.^17,18^ In addition, they enhance osteoblastic differentiation through bone morphogenic protein 2 (BMP-2).^19^ In the treatment of periodontitis, statins are particularly valuable due to their anti-inflammatory and osteogenic properties, which can positively impact managing this condition.^20,21^ Thus, to preserve tooth sockets, this trial evaluated the efficacy of topical lovastatin and synthetic bone grafting material (nano-crystalline hydroxyapatite). It also made a histomorphometric evaluation of the treatment areas.

## Methods

###  Data Source

**Figure 1:**
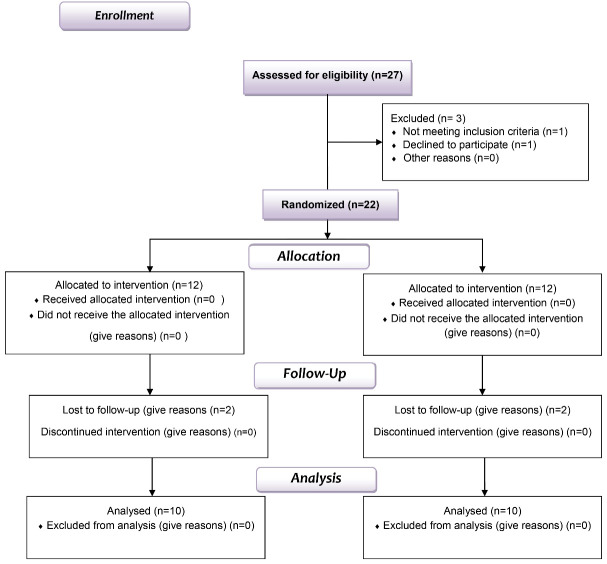
Flow chart of the study design

 In a randomized, double-blind clinical trial, we recruited 20 (8 men and 12 women, 24‒53 years of age) male and female patients from the outpatient section of the Department of Periodontology at the Broujerd Islamic Azad University of Medical Sciences, Faculty of Dentistry, Lorestan, Iran. These patients were monitored and followed for over 9 months. Initially, the research was carried out in strict adherence to the ethical guidelines outlined in the Declaration of Helsinki (2008 version). We obtained ethical approval from the institutional ethics committee and review board of the Islamic Azad University Medical Sciences, and all the participants provided written informed consent before inclusion in the study. The study was registered on clinicaltrials.gov under the ID NCT03981601. The study design is depicted in the CONSORT flowchart (Figure 1).

###  Patient Selection 

 Twenty healthy participants were selected for this study, each having two single teeth in the anterior region of the maxilla that required extraction and subsequent rehabilitation with dental implants. The participants were enrolled in ascending order, and a researcher not involved in any other aspect of the study randomly assigned them to either the control group or one of the two test groups using a computer-generated randomization list, maintaining a 1:1 ratio. The recruitment of volunteers spanned over six months, and all the participants were monitored for 12 months following prosthetic rehabilitation. The assignment of participants to the test groups was further facilitated by an envelope distribution system managed by the principal investigator. The recruitment was eligible for this study according to specific exclusion and inclusion criteria, which are outlined below.

###  Inclusion Criteria

 The participants were required to be in good overall health, at least 24 years old, and possess a minimum of 20 teeth. They needed to demonstrate good oral hygiene, indicated by a plaque index and bleeding on probing of ≤ 20%. Additionally, the candidates required socket preservation in two intact sites in the anterior maxilla due to various dental issues, such as endodontic failures, caries, and root or tooth fracture.

###  Exclusion Criteria

 Individuals were excluded from the study if they had any systemic health issues, were pregnant or breastfeeding, were smokers, had ankylosed tooth, had a history of radiotherapy or chemotherapy for malignancies within the last five years, exhibited a plaque index and gingival bleeding index > 25%, had undergone periodontal surgery in the desired areas in the past six months, had an active infection, extensive caries, used removable prostheses, had periodontal disease and soft tissue recession, had orthodontic treatment in their history, suffered from allergies to lovastatin, experienced tooth ankylosis, or had fenestration or dehiscence around the tooth socket.

###  Primary and Secondary Outcomes 

 The primary outcome of this study was to promote healing in the affected area while also reducing postoperative pain and swelling. In addition, the secondary outcome involved assessing and comparing the histological, clinical, and radiographic consequences of tooth socket preservation following extraction, using synthetic nano-crystalline hydroxyapatite bone graft, with and without the addition of lovastatin.

###  Sample Size

 A straightforward sampling method was employed, adhering to the predefined inclusion and exclusion criteria. Based on the findings of a study by Graziani et al.,^22^ and using the paired sample difference t-test option of Pass 11 software, with an α level set at 0.05 and a β level at 0.2, the mean standard deviation was determined to be 16. Moreover, with a minimum significant difference established at 15%, the estimated minimum sample size required was 20 samples in two groups.

###  Statistical Methods and Data Analysis

 To analyze clinical indicators at different time intervals, we used a repeated-measures ANOVA model, incorporating two repeated variables: time and type of intervention. For the histomorphometry variables, given the likelihood of non-normal distribution, the Wilcoxon signed-rank test or its parametric equivalent was applied following the normality assessment. Histological variables were also compared using the Wilcoxon signed-rank test (*P* < 0.05).

###  Presurgical Procedures 

 We systematically reviewed the medical and dental histories of each patient, followed by a thorough evaluation using periapical radiographs, clinical photographs, and study casts, along with clinical examinations of the extraction sites. After this assessment, the volunteers received comprehensive instructions on oral hygiene. The study casts will be used in the reentry procedures to ensure precise bone biopsy collection from the grafted sockets’ center. All the patients were informed of the treatment plan and the study’s objectives, and they underwent atraumatic extraction in both of their upper anterior regions in a single visit.

###  Surgical Procedure 

**Figure 2:**
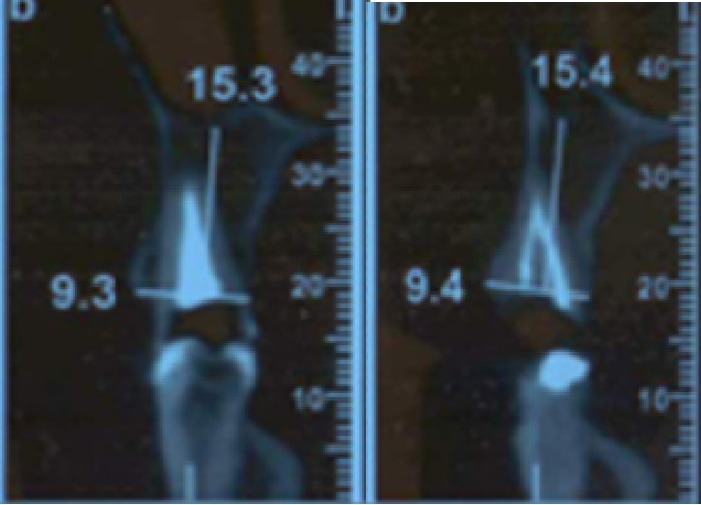
Primary radiographs

**Figure 3:**
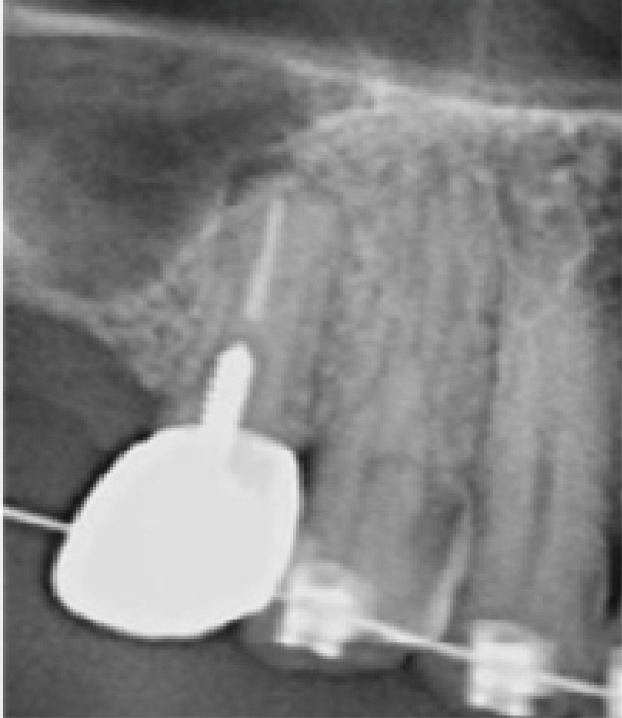
Primary radiograph

**Figure 4:**
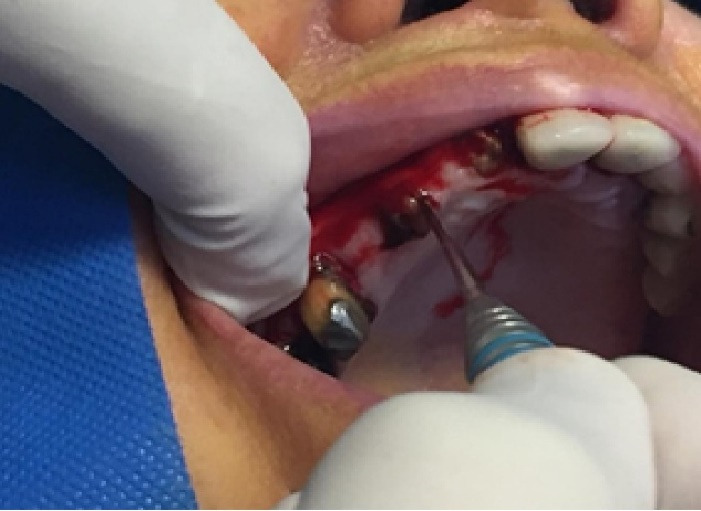
Extracting a tooth with a periotome

**Figure 5:**
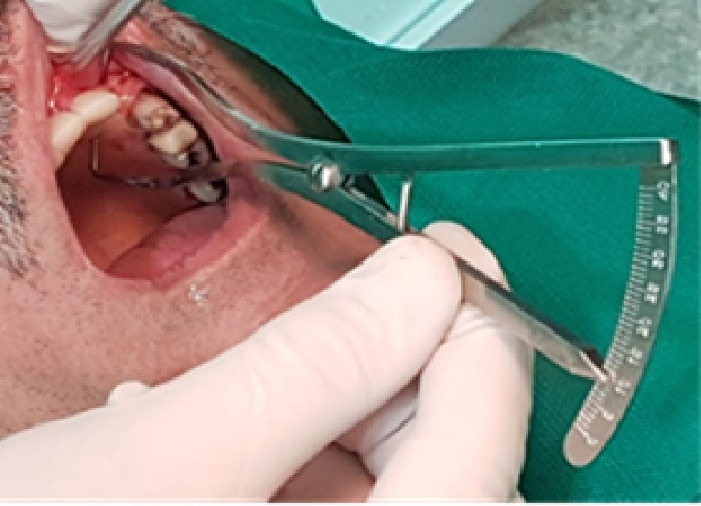
Buccolingual width measurement

**Figure 6:**
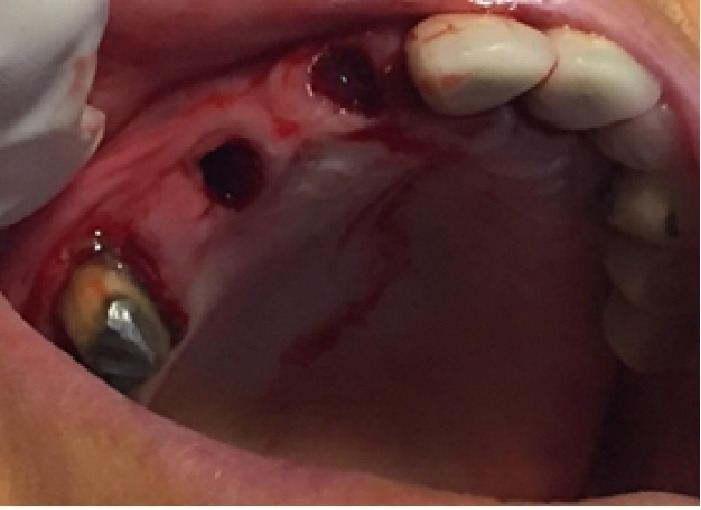
The alveolar socket was then carefully irrigated with normal saline and an 0.2% chlorhexidine solution for 30 seconds

**Figure 7:**
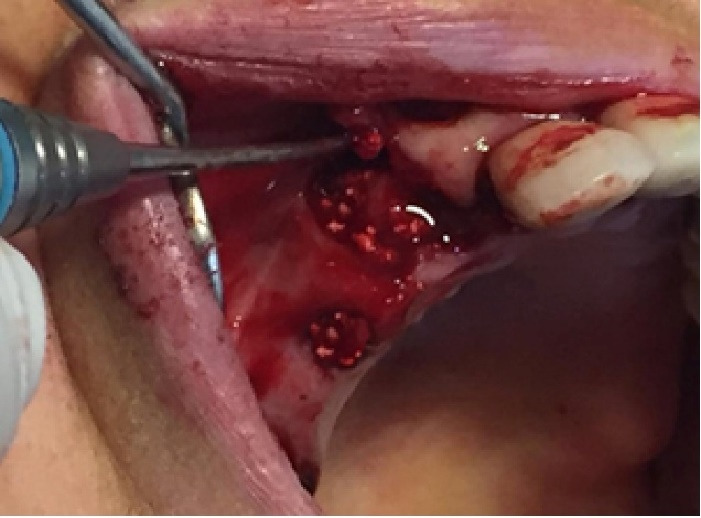
Socket preservation with bone material with and without lovastatin

**Figure 8:**
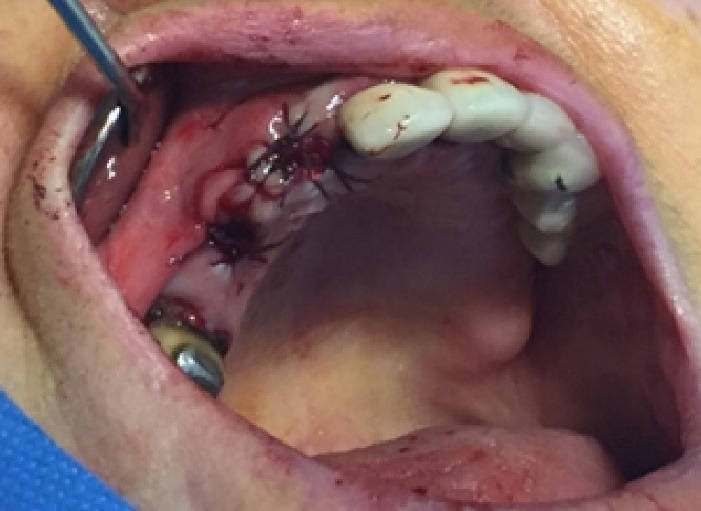
Suturing (Figure 8 and horizontal mattress)

 In this study, we recruited 20 patients needing the extraction of two anterior teeth. CBCT imaging was completed for the initial preoperative assessment ahead of tooth extraction. (Figures 2 and 3). Also, a periodontist carried out the extractions using a minimally invasive technique under local anesthesia with lidocaine containing 1:100,000 epinephrine. The procedure did not require a flap and used both a periotome and an elevator (Figure 4). Careful removal of residual granulation tissue, cysts, or lesions was performed using a curette, taking special care to avoid the buccal plane area to preserve the remaining bundle bone and prevent further complications with the buccal bone. To assess the width of the cavity—the distance between the buccal and palatal bone walls—we employed a bone caliper (Figure 5). The intra-lingual/facial epithelium was effectively removed using a bur, followed by thorough irrigation of the alveolar socket with normal saline and an 0.2% chlorhexidine solution for 30 seconds (Figure 6). In the maxilla, we randomly selected two sites for the placement of the graft material. The packages for the drug and placebo were identical in appearance and labeled as A and B, while the investigator remained blinded to the contents of the packages. The same dose was used in both packages. One site was identified as group A; we used 10 mg of lovastatin (Pursina Pharmaceutical Co., Tehran, Iran), to blend with hydroxyapatite (Nanobone, granule size: 0.6 mm, Artoss, Rostock, Germany), which consisted of synthetic nano-crystalline hydroxyapatite. In contrast, group B consisted solely of Nanobone, which filled the sockets created by the tooth extractions (Figure 7). It is noteworthy that the graft material was stored in sterile distilled water for at least 10 minutes before its application into the sockets. Once the tooth socket was filled with the graft material, either with or without medication, it was gently compacted with a condenser. An absorbable collagen cone (Dentegris Co., Germany) was then placed over the socket. To enhance tensile strength and durability, silk sutures were applied to securely close the wound edges, using Figure 8 suture patterns and horizontal mattress sutures (Figure 8).

###  Postoperative Care

 For effective plaque control, the patients were instructed to use a 0.12% chlorhexidine gluconate mouthwash twice daily for two weeks after surgery. Additionally, as part of their postoperative care, they received amoxicillin (500 mg) three times a day for 5 days, along with ibuprofen (400 mg) every 6 hours, taken with food or drink, to prevent infection, alleviate pain, and reduce swelling. After 2 weeks, it was advised that patients apply 3% tetracycline ointment twice daily to the affected areas using a swab for another 2 weeks. During the initial recovery period, a liquid diet was recommended, gradually transitioning to soft foods. To avoid food particles entering the incision site, chewing food on one side was recommended. This care regimen was applied to both the extraction and socket preservation procedures. Sutures were removed 2 weeks after surgery, and 6 months later, the surgical sites were reopened for implant insertion.

###  Surgical Reentry 

**Figure 9:**
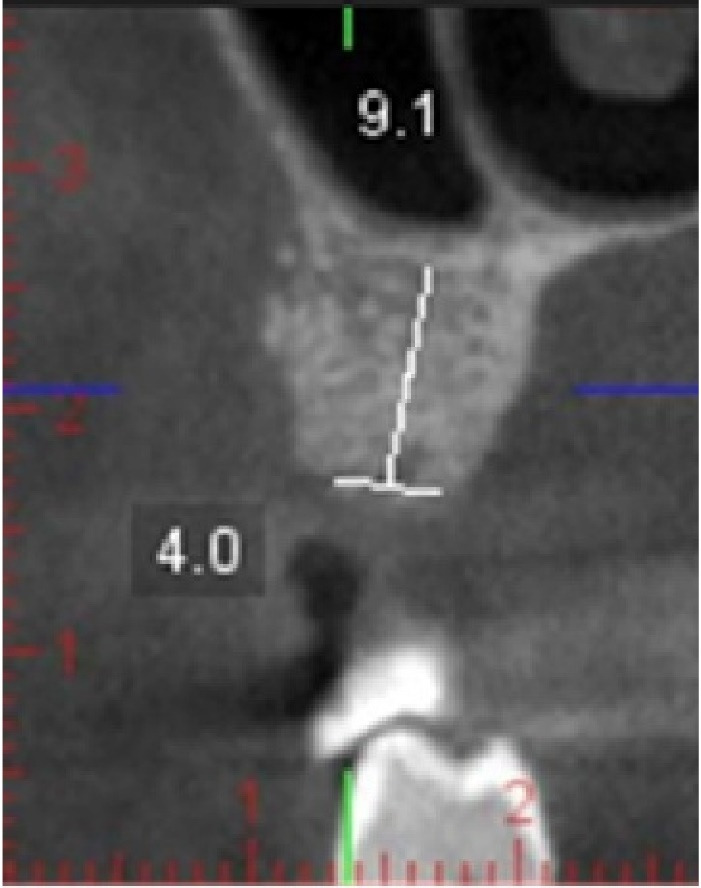
CBCT after six months

**Figure 10:**
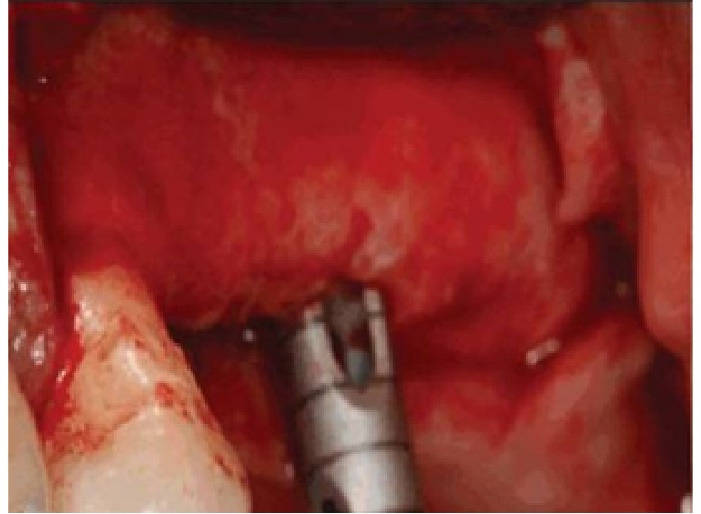
To acquire a bone sample (biopsy) after 6 months, utilizing a trephine

**Figure 11:**
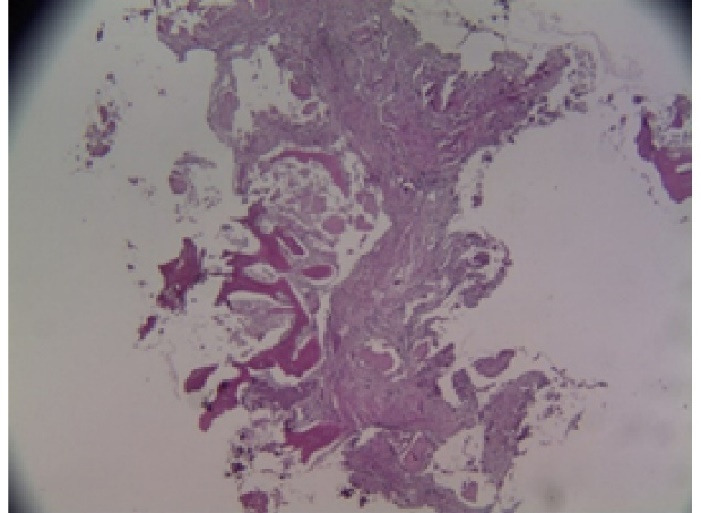
Guide pin during implant drilling after 6 months

 The implants were positioned 24 weeks after extraction. A mucoperiosteal flap was created to access the underlying tissue. Using a caliper again, the horizontal ridge width was measured buccolingually. For further evaluation, a CBCT scan was taken after treatment (Figure 9). A core biopsy was taken from the center of the extraction site, reaching a depth of 6 mm. This was performed with a trephine bur measuring 3.4 mm in diameter to gather the biopsy specimen (Figure 10). Following this procedure, dental implants were inserted following the manufacturer’s surgical guidelines. After 6 months, all the implants were successfully placed at both the control and test sites (Figure 11).

###  Histologic/ Histomorphometric Evaluation

 After delivery to the laboratory and sample coding, they were fixed in 10% formalin for 48 hours. Following this stage, they were placed in 10% formic acid for decalcification for 4 days. Then, the samples were washed in running water, and paraffin blocks were prepared. Subsequently, 5-µm-thick sections were cut to achieve the highest length and diameter of the tissue. The sections were prepared and evaluated for histological examination and histomorphometric analysis using H&E staining by Dr. Mashhadi Abbas at the School of Dentistry, Shahid Beheshti University, in a blind manner. Histological evaluation of the samples was performed at × 40, × 100, and × 400 magnifications using the following scores:

1. Inflammation: Acute and chronic types were scored as follows:
  A: score 0: < 10%, score B: 10–30%, score C: 30–50%, and score D: 50% or more
2. Foreign body reaction or remodeling: The presence of multinucleated giant cells
3. Histomorphometric analysis: Images were captured from the samples using a Motic microscope at × 40 magnification. Then, bone formation percentage was measured using HMMA software (ver. 1.1/SBMU/Iran).

## Results

 In this study, we assessed 20 patients, including 12 females and 8 males, with an average age of 44 ± 1.70 years. Throughout the research, 2 of the 4 patients in each group chose to withdraw from the evaluations and treatments for various reasons. Ultimately, 20 patients completed the study in each group. All the participants reported uneventful recovery during their follow-up, with no severe pain, significant swelling, hypersensitivity issues, or adverse side effects. Furthermore, patients did not express any discomfort or specific concerns during examinations throughout the treatment period. Following the clinical protocol, each patient underwent a designated treatment, and the study advanced without any postoperative complications. We analyzed 20 histological samples to assess factors related to bone metabolism and vascularization. Microscopic examinations at a magnification of × 100 revealed mineralized areas of newly formed bone distributed throughout the specimens in the group with lovastatin mixed with Nanobone, which also showed notably well-differentiated capillary vascularization. In contrast, the group with lovastatin mixed with Nanobone exhibited no evidence of acute or chronic inflammatory infiltration in any sections. Meanwhile, samples from the Nanobone group displayed some inflammatory cells, mainly consisting of mononuclear cells, such as lymphocytes and macrophages, suggesting an active inflammatory or immune response. The descriptive data indicated that the combined material usage resulted in slightly higher values. We thoroughly evaluated all the samples from the Nanobone group. Statistical analyses conducted using the Mann-Whitney U and Wilcoxon W tests revealed significant differences in bone formation levels between the Nanobone group (average of 51.6944%) and the Nanobone + 10-mg lovastatin group (average of 60.7956%) (*P* < 0.05), highlighting lovastatin’s role in promoting bone formation (Table 1). Moreover, the examination of residual bonding materials showed a significant difference between the Nanobone group (average of 1.7078 mm) and the Nanobone + lovastatin group (average of 0.4844 mm), indicating that lovastatin significantly reduces the amount of residual bonding materials (Table 2). Clinical analyses also revealed that the Nanobone group averaged 0.889 mm, while the Nanobone + lovastatin group averaged 0.589 mm (*P* = 0.05), suggesting that lovastatin significantly impacted ridge width reduction (Table 3) (*P* < 0.05).

 Notably, laboratory samples showed no signs of inflammation or foreign body reactions based on the established criteria. When analyzing bone inflammation in groups A and B, the P value was determined to be 1.000 (*P* ≥ 0.05). Although the lovastatin group showed a lower level of bone inflammation compared to the bone powder group alone, these differences were not statistically significant (Table 4). Additionally, when assessing the response of the remaining graft material in group A versus group B, the *P* value was recorded at 0.721 (*P* ≥ 0.05). The lovastatin group demonstrated a reduced reaction of the remaining graft material compared to the bone powder group, but, similarly, these results were not statistically significant (Table 5). In group A, the connective tissue appeared normal, while in group B, there was evidence of granulation tissue and fibrosis. Furthermore, group B had a higher incidence of bleeding compared to group A. No statistically significant difference was found in the frequency of foreign body reactions between the two groups. Hence, the results indicate that while Nanobone alone effectively supported ridge preservation, its performance fell short compared to the combination of Nanobone and lovastatin. A key strength of this study is the histological analysis conducted on human biopsy specimens, which differentiates it from most related studies that typically rely on animal models. Additionally, the relatively short follow-up period of 6 months can be viewed as a strength, though further research is necessary. Future studies should include a larger sample size and extend the follow-up duration to better assess the long-term outcomes of tooth cavity preservation when using lovastatin. Overall, while both treatment groups demonstrated promising efficacy in ridge preservation, the combination of Nanobone and lovastatin yielded superior histological, clinical, and radiographic results compared to Nanobone alone.

**Table 1:** The level of bone formation in groups A and B

**Bone formation**	**No**	**Minimum**	**Maximum**	**Mean±SD**	***P*** ** value**
Group A: (Lovastatin)	10	10.25	80.25	51.6944 ± 25.47244	0.436
Group B: (Nanobone)	10	84.71	24.19	60.7956 ± 21.66420	

**Table 2:** The level of residual bonding materials in groups A and B

**Residual bonding materials**	**No**	**Minimum**	**Maximum**	**Mean±SD**	***P*** ** value**
Group A: (Lovastatin)	10	.00	4.00	1.7078 ± 1.53068	0.049
Group B: (Nanobone)	10	.00	2.30	0.4844 ± 0.81405	

**Table 3:** The extent of changes in the alveolar bone width in groups A and B

**Ridge width**	**No**	**Minimum**	**Maximum**	**Mean±SD**	***P*** ** value**
Group A: (Lovastatin)	10	0.5	0.7	0.589 ± .0782	0.001
Group B: (Nanobone)	10	0.8	1.0	0.889 ± 0.0782	

**Table 4:** The degree of inflammation present in the bone developed in the treated regions across the various study groups

**Inflammation **	**No**	**0‒10**	**10‒30**	**30‒50**	***P*** ** value**
Group A: (Lovastatin)	10	62.5	25	12.5	1.000
Group B: (Nanobone)	10	65.5	25	12.5	

**Table 5:** The rate at which the excess graft material contributes to the foreign body response in groups A and B

**Foreign body reaction**	**No**	**Positive**	**Negative**	***P*** ** value**
**Group A: (Lovastatin)**	10	12.5	87.5	0.721
**Group B: (Nanobone)**	10	25	75	

###  Histomorphometry Results

**Figure 12:**
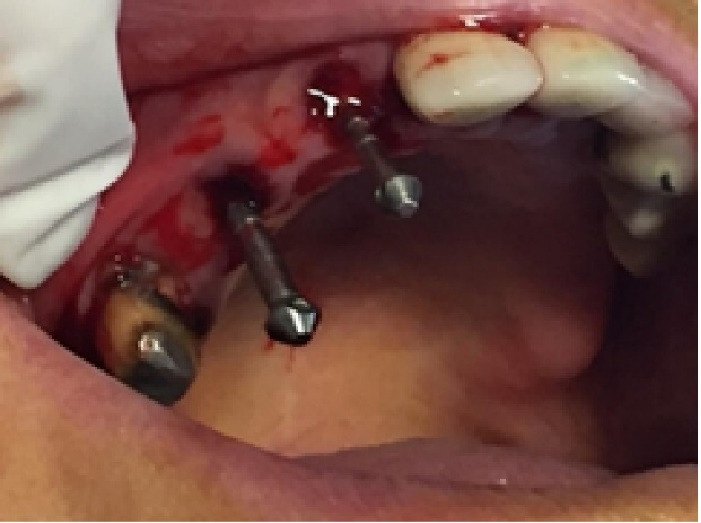
Fibrous tissue encased in remnants of material has undergone ossification in the lovastatin group, revealing the presence of bone trabeculae ( × 40)

Figure 12. Fibrous tissue encased in remnants of material has undergone ossification in the lovastatin group, revealing the presence of bone trabeculae ( × 40).

**Figure 13:**
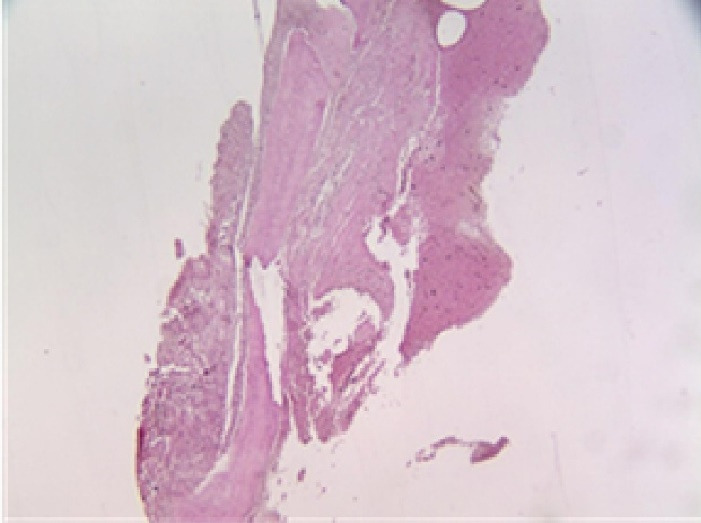
Fibrous tissue containing residual material (represented as empty spaces) alongside newly formed bone in the Nanobone group at a magnification of × 40

Figure 13. Fibrous tissue containing residual material (represented as empty spaces) alongside newly formed bone in the Nanobone group at a magnification of × 40.

## Discussion

 After tooth extraction, it is common for the surrounding alveolar bone to experience resorption, which can affect the periodontal tissue. We often notice partial bone defects in areas lacking teeth, and this typically necessitates guided bone regeneration to help restore the alveolar bone.^23,24^ Many studies, both preclinical and clinical, have demonstrated that applying guided bone regeneration (GBR) on exposed implant surfaces can lead to effective bone integration and help preserve alveolar bone volume. Research also shows that the survival rates for implants placed simultaneously with GBR are comparable to those for implants placed in intact alveolar bone without GBR.^25,26^ However, autologous bone grafting remains the gold standard for reconstructing bone defects, although it has limitations, such as the availability of bone volume and the need for additional surgical sites.^27^ To overcome these challenges, several alternative materials have been developed, including allogeneic bone, xenogeneic bone, and synthetic bone, with ongoing research aiming to discover even better options. Among these, synthetic bone is becoming increasingly popular due to its advantages, including ease of mass production, cost-effectiveness, and a lower risk of disease transmission.^28,29^ This research study specifically evaluated the histomorphometry of nano-crystalline hydroxyapatite graft material in socket preservation surgery, both with and without the inclusion of 10 mg of lovastatin.

 Conducted as a double-blind, randomized trial, this study ensured unbiased results and obtained informed consent from all the participating patients. In total, 40 sites were examined, randomly divided into two groups. One group received the synthetic bone graft substitute HA (Nanobone) graft material alongside lovastatin, while the other group received only the synthetic bone graft substitute HA. The results indicated that adding lovastatin significantly enhanced bone formation in dental sockets by 9%.

 Similar studies on animal models have also corroborated this positive effect.^30‒32^ Ayukawa et al.^33^ investigated the role of statins in bone healing and found that by day 5, there was a significant boost in bone formation linked to statin use.^33^ Similarly, Rajeshwari et al.^20^ focused on patients suffering from chronic periodontitis, who were also smokers, examining the localized use of simvastatin. Their findings indicated impressive improvements in key metrics such as the bleeding index (BI), probing depth (PD), and clinical attachment level (CAL) over the course of 3, 6, and 9 months. Additionally, radiographic assessments at the 6 and 9-month intervals revealed advancements in bone defects, evaluated through computer software analysis. This research underscores simvastatin’s effectiveness in enhancing bone healing as opposed to a placebo, reinforcing the favorable impact of statins on bone formation.^20^

 On another front, Tanabe et al.^34^ examined the combination of fluvastatin with biodegradable gelatin hydrogel in circular bone defects in 15-week-old mice, reporting a significant enhancement in bone formation in comparison to a placebo.^34^ Morris et al.^35^ evaluated the effects of local simvastatin injections on the healing of three-walled intra-bony and furcal defects in beagle dogs. Their results indicated a significant enhancement in ridge augmentation and new cementum formation in the dogs treated with simvastatin compared to those in the control group.^35^ Furthermore, Wu et al.^36^ found that the residual alveolar ridge was considerably taller in the experimental group relative to the control group. They also noted a significant rise in bone mineral density among the treated dogs. By the four-week interval, the experimental group exhibited a larger area of newly formed bone, along with a better rate and quality of bone formation at various assessment points, except for the one-week evaluation. These observations imply that local application of simvastatin can effectively preserve the residual alveolar bone by promoting bone formation in the extraction socket.^36^

 Sezavar et al.^37^ also confirmed that simvastatin positively influenced bone formation after two months, based on their examination of 20 human sockets.^37^ Wong and Rabie^31^ conducted a thorough quantitative analysis on 100 sections to assess new bone formation through image analysis. Their results were impressive: defects treated with statin collagen grafts exhibited a significant increase of 308% in new bone compared to those treated with collagen grafts alone. Interestingly, no bone formation was noted in the passive control group. This suggests that statin collagen grafts possess osteoinductive properties, making them promising materials for bone grafting procedures.^31^ In addition, research highlights the importance of non-bone materials like synthetic nano-crystalline hydroxyapatite and silica, which are produced via the sol-gel process, in bone regeneration. Silica gel, in particular, facilitates collagen and bone formation.^38^ Furthermore, Soliman et al.^39^ reported that the combination of simvastatin led to a modest increase in the mineralized area of newly formed bone, alongside a notable presence of well-differentiated capillary vascular formations. Hence, local application of lovastatin has been shown to promote bone formation in extraction sockets. This method is straightforward and offers a cost-effective solution for accelerating bone regeneration after tooth extraction. Nonetheless, more extensive studies with larger sample sizes and longer follow-up periods are required to reach valid conclusions. Additionally, further investigation is necessary to understand how bone formation varies at different anatomic sites with the local application of simvastatin. The potential of statins as a local treatment for regenerating bone defects is promising, given their observed osteogenic and angiogenic properties. To establish their effectiveness, more randomized clinical trials with larger patient populations and histological analyses are essential.

## Conclusion

 Lovastatin, when applied topically, is highly effective in promoting bone formation, minimizing the reduction in ridge width, and reducing the amount of remaining graft material. Importantly, this treatment does not lead to any inflammation or foreign body reactions, as indicated by the findings of this study.

## Competing Interests

 The authors declare that they have no competing interests related to the authorship or publication of this study.

## Data Availability

 Once published, individuals interested in obtaining the raw or processed data needed to replicate these findings can contact the corresponding author.

## Ethical Approval

 The Human Research Ethics Committee at Borujerd of the Islamic Azad University Medical Sciences in Lorestan, Iran, approved the research for this study, under code 62038.
